# Melatonin Alleviates Liver Mitochondrial Dysfunction in Leptin-Deficient Mice

**DOI:** 10.3390/ijms25168677

**Published:** 2024-08-08

**Authors:** Beatriz de Luxán-Delgado, Yaiza Potes, Adrian Rubio-González, Juan José Solano, José Antonio Boga, Eduardo Antuña, Cristina Cachán-Vega, Juan Carlos Bermejo-Millo, Nerea Menéndez-Coto, Claudia García-González, Gonçalo C. Pereira, Beatriz Caballero, Ana Coto-Montes, Ignacio Vega-Naredo

**Affiliations:** 1 Department of Morphology and Cell Biology, University of Oviedo, Julián Clavería s/n, 33006 Oviedo, Spain; b.luxandelgado@gmail.com (B.d.L.-D.); potesyaiza@uniovi.es (Y.P.); adrianrubiogonzalez@gmail.com (A.R.-G.); edugan.97@gmail.com (E.A.); bermejomillo@gmail.com (J.C.B.-M.); mdezcotonerea.fuo@uniovi.es (N.M.-C.); caballerobeatriz@uniovi.es (B.C.); acoto@uniovi.es (A.C.-M.); 2 Instituto de Investigación Sanitaria del Principado de Asturias (ISPA), Av. Del Hospital Universitario, 33011 Oviedo, Spain; solano.jaurrieta@gmail.com (J.J.S.); joseantonio.boga@sespa.es (J.A.B.);; 3 Instituto de Neurociencias del Principado de Asturias (INEUROPA), University of Oviedo, Julián Clavería s/n, 33006 Oviedo, Spain; 4 Geriatrics Service, Monte Naranco Hospital, 33012 Oviedo, Spain; 5 Microbiology Department, Hospital Universitario Central de Asturias, Avenida de Roma s/n, 33011 Oviedo, Spain; 6 School of Biochemistry, Medical Sciences Building, University of Bristol, Bristol BS8 1TD, UK; goncalocpereira@gmail.com

**Keywords:** obesity, leptin deficiency, melatonin, liver, mitochondrial dysfunction, adipogenesis

## Abstract

Despite efforts to elucidate the cellular adaptations induced by obesity, cellular bioenergetics is currently considered a crucial target. New strategies to delay the onset of the hazardous adaptations induced by obesity are needed. Therefore, we evaluated the effects of 4 weeks of melatonin treatment on mitochondrial function and lipid metabolism in the livers of leptin-deficient mice. Our results revealed that the absence of leptin increased lipid storage in the liver and induced significant mitochondrial alterations, which were ultimately responsible for defective ATP production and reactive oxygen species overproduction. Moreover, leptin deficiency promoted mitochondrial biogenesis, fusion, and outer membrane permeabilization. Melatonin treatment reduced the bioenergetic deficit found in ob/ob mice, alleviating some mitochondrial alterations in the electron transport chain machinery, biogenesis, dynamics, respiration, ATP production, and mitochondrial outer membrane permeabilization. Given the role of melatonin in maintaining mitochondrial homeostasis, it could be used as a therapeutic agent against adipogenic steatosis.

## 1. Introduction

Mitochondrial dysfunction, which is characterized by a reduction in the number and/or function of mitochondria, is implicated in a myriad of disorders, ranging from neurodegenerative and cardiovascular diseases to skeletal muscle disorders, sepsis, and psychiatric conditions [1]. In addition to its primary role in ATP synthesis, mitochondrial (dys)function is also a key driver of the aging process [2]. As the main powerhouses of the cell, mitochondria are crucial for lipid metabolism and maintaining cellular homeostasis [3]. In fact, disrupted mitochondrial function is linked to obesity and other metabolic disorders [1].

Mitochondrial activity, biogenesis, and dynamics are central to the adipogenic process and adipocyte differentiation [4,5]. As adipose tissue expands with obesity, free fatty acids migrate to the liver, leading to hepatic steatosis, the main liver-related consequence of obesity [6]. The liver responds by increasing lipid droplet biogenesis [7], but hepatic steatosis can also result from hepatocytes transforming into adipocytes, a process known as adipogenic steatosis [8]. Other contributing factors include the upregulation of lipogenic pathways and the inhibition of triglyceride secretion [9].

Several studies have highlighted the essential role of mitochondrial dysfunction in the development of hepatic steatosis [10,11,12]. Excessive fat in the liver affects mitochondria because an overload of the electron transport chain (ETC) leads to defective mitochondrial fatty acid oxidation with subsequent lipotoxicity. These conditions induce specific events related to mitochondrial quality, such as increased mitochondrial reactive oxygen species (ROS) production and mitochondrial DNA (mtDNA) damage, as well as reduced mitochondrial biogenesis, mtDNA copy numbers, mtDNA repair, mitochondrial fusion and mitochondrial degradation by autophagy [13,14]. This failure of mitochondrial quality control impairs mitochondrial function [15] and reduces ATP levels [16], ultimately triggering mitochondrial pathways of cell death [17]. Consequently, excessive lipid deposits in the liver cause mitochondrial stress and dysfunction, disrupting lipid and glucose metabolism and leading to obesity-related issues, such as hypertriglyceridemia, insulin resistance and diabetes [18].

Melatonin, a powerful natural antioxidant, is well known for its hormonal role in the regulation of circadian rhythms [19]; however, it also plays roles in body weight control [20], adipogenesis [21], insulin resistance, diabetes [22], inflammation [23], glucose and lipid metabolism [24] and hepatic steatosis [25]. Melatonin deficiency has been repeatedly linked to obesity [26,27,28]. Given the ability of mitochondria to accumulate melatonin, numerous studies suggest that melatonin helps to regulate mitochondrial homeostasis [29,30]. This mechanism makes melatonin a key focus in obesity-related mitochondrial function research [11,31].

Hormones such as insulin and leptin, which are involved in energy homeostasis, also follow circadian rhythms, indicating a close relationship with mitochondrial bioenergetics [32]. Leptin, the primary adipokine from white adipose tissue, signals nutritional status by decreasing appetite, inhibiting food intake, and stimulating energy expenditure [33,34]. Leptin deficiency leads to an uncontrolled appetite and hepatic dysregulation of glucose and fat metabolism. Consequently, leptin-deficient (ob/ob) mice are widely used to study obesity [35] and related hepatic complications [11,31,36,37].

In light of this extensive research, the aim of the present study was to elucidate the effects of obesity on lipid metabolism and mitochondrial function in the livers of leptin-deficient mice while assessing the therapeutic potential of melatonin.

## 2. Results

### 2.1. Melatonin Attenuates Lipid Storage in the Livers of ob/ob Mice

**Figure 1:**
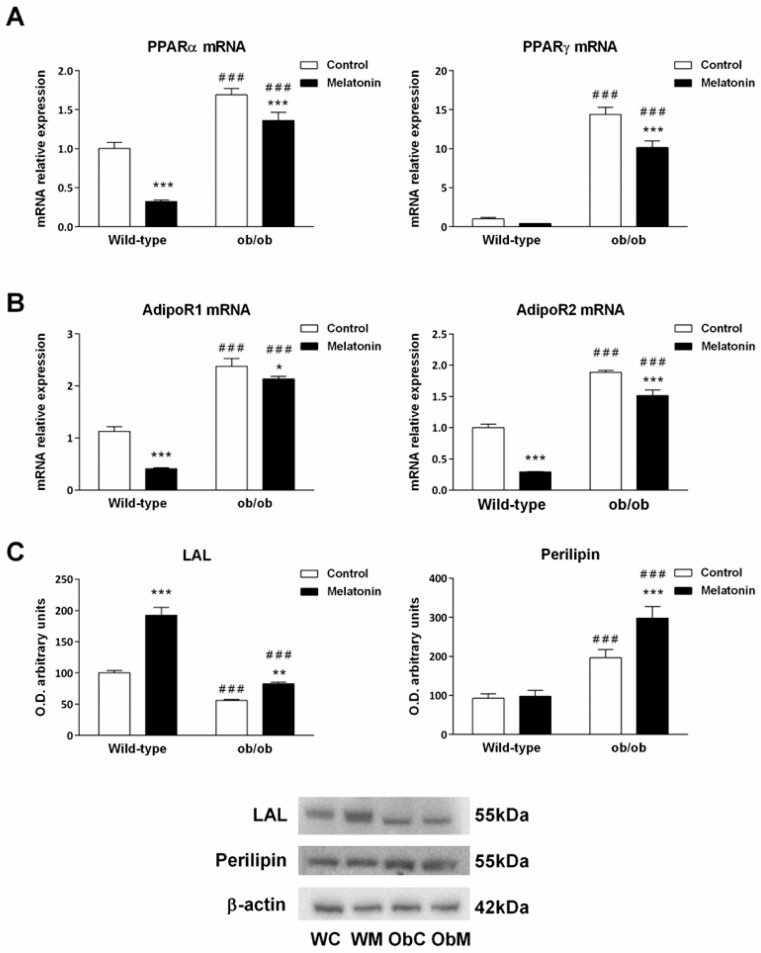
Adipogenic, lipogenic, adiponectin-related, and lipid droplet markers from the livers of wild-type and ob/ob mice. (**A**) mRNA expression of peroxisome proliferator-activated receptor alpha (PPARα) and gamma (PPARγ). (**B**) mRNA expression of adiponectin receptors (AdipoR1 and AdipoR2). (**C**) Bar chart showing the semiquantitative optical density (arbitrary units of blot bands) and Western blot results of lysosomal acid lipase (LAL) and perilipin protein expression normalized to β-actin. The data are expressed as the means ± SDs, which were calculated from at least three independent measurements. WC—untreated wild-type; WM—wild-type plus melatonin; ObC—untreated ob/ob; ObM—ob/ob plus melatonin. Statistical comparisons: # wild-type vs. ob/ob and * treated with melatonin vs. untreated counterpart. The number of symbols indicates the level of statistical significance: * for *p <* 0.050, ** for *p <* 0.010, and ***/### for *p <* 0.001.

We first evaluated the expression levels of genes involved in lipid storage, metabolism and adipogenesis, such as PPARγ (peroxisome proliferator-activated receptor gamma) and PPARα (peroxisome proliferator-activated receptor alpha), in the livers of healthy and obese animals [38,39,40]. Our results showed that livers from leptin-deficient mice expressed significantly higher levels of both PPARγ (15-fold) and PPARα mRNA (>1.5-fold), suggesting that ob/ob mice exhibited increased hepatic adipogenesis and steatosis. Melatonin treatment for 4 weeks decreased PPARα mRNA levels in wild-type mice and both PPARα and PPARγ mRNA levels in ob/ob animals (*p* < 0.001), albeit not to control levels (Figure 1A). Next, we studied the mRNA expression of adiponectin receptors 1 and 2 (AdipoR1 and AdipoR2), which are involved in preadipocyte differentiation, lipid content and insulin response [41]. Compared with their wild-type counterparts, leptin-deficient animals presented higher expression levels of both receptors (*p* < 0.001), whereas melatonin caused a significant decrease in their mRNA expression in both genotypes (*p <* 0.050) (Figure 1B).

Lysosomal acid lipase (LAL) and perilipin are proteins that participate in intracellular lipid metabolism. Consistent with their obese phenotype, we found that livers from ob/ob animals presented a significant decrease in LAL levels and an increase in perilipin content compared with those of wild-type mice (*p* < 0.001). Melatonin treatment led to an increase in LAL expression in both strains (*p* < 0.010), but perilipin increased LAL expression only in ob/ob mice (*p* < 0.001) (Figure 1C). These findings suggest that melatonin generally induces the hydrolysis of triglycerides and cholesteryl esters.

**Figure 2:**
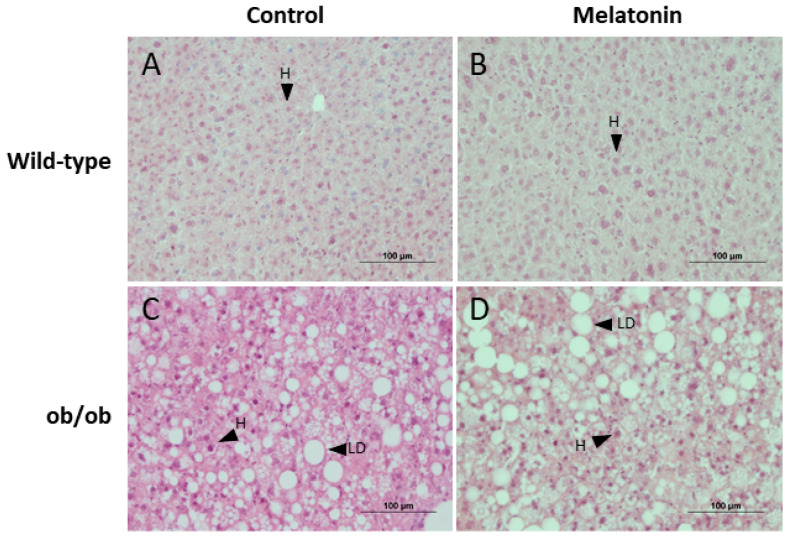
Histological analysis of livers from wild-type and ob/ob mice. Light microscopy images of hematoxylin–eosin-stained sections of livers from (**A**) wild-type mice, (**B**) wild-type mice treated with melatonin, (**C**) ob/ob mice, and (**D**) ob/ob mice treated with melatonin. Scale bar: 100 μm. LD—lipid droplet; H—hepatocyte.

Morphological analysis of the liver revealed greater lipid droplet deposition, and therefore hepatic steatosis, in ob/ob mice than in wild-type mice. However, no histological differences were noted after melatonin treatment (Figure 2).

Taken together, our results indicate that leptin deficiency leads to an increase in lipid storage and adipogenesis in the liver, which can be improved by melatonin treatment.

### 2.2. Melatonin Counteracts Leptin Deficiency-Induced Mitochondrial Biogenesis

**Figure 3:**
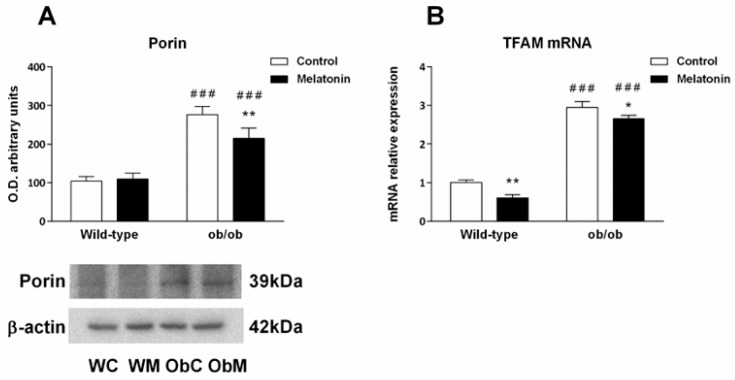
Mitochondrial mass and biogenesis markers in the livers of wild-type and ob/ob mice. (**A**) Bar charts showing the semiquantitative optical density (arbitrary units of blot bands) of porin normalized to β-actin. (**B**) mRNA expression of mitochondrial transcription factor A (TFAM), an indicator of mitochondrial biogenesis. The data are expressed as the means ± SDs that were calculated from at least three separate measurements. WC—untreated wild-type; WM—wild-type plus melatonin; ObC—untreated ob/ob; ObM—ob/ob plus melatonin. Statistical comparisons: # wild-type vs. ob/ob and * treated with melatonin vs. untreated counterpart. The number of symbols indicates the level of statistical significance: * for *p <* 0.050, ** for *p <* 0.010, and ### for *p <* 0.001.

Mitochondrial mass was evaluated indirectly by measuring the levels of Porin, a mitochondrial housekeeping protein [42], and the levels of mitochondrial transcription factor A (TFAM), a key activator of mitochondrial DNA transcription that participates in mitochondrial genome replication. As shown in Figure 3A, leptin deficiency increased in Porin levels, suggesting an increase in mitochondrial mass. Similarly, TFAM mRNA levels in the livers of ob/ob mice were increased by approximately threefold compared with those in the livers of wild-type mice (Figure 3B). In both cases, melatonin treatment partially prevented leptin-induced effects.

Overall, mitochondrial biogenesis seems to be triggered in leptin-deficient mice, whereas melatonin treatment counteracts this process in both genotypes.

### 2.3. Melatonin Reverses the Remodeling of the ETC/OXPHOS Machinery Induced by Leptin Deficiency

Considering the above-reported effects on mitochondrial biogenesis, we next analyzed the expression of the subunits of the mitochondrial ETC complexes. A total of five subunits, one for each ETC complex, were evaluated namely, NADH dehydrogenase (ubiquinone) 1 b subcomplex 8 (NDUFB8) from complex I; succinate dehydrogenase (ubiquinone) iron–sulfur subunit (SDHB) from complex II; ubiquinol–cytochrome c reductase core protein II (UQCRC2) from complex III; cytochrome c oxidase subunit I (MTCO1) from complex IV and ATP synthase subunit α (ATP5A) from complex V.

**Figure 4:**
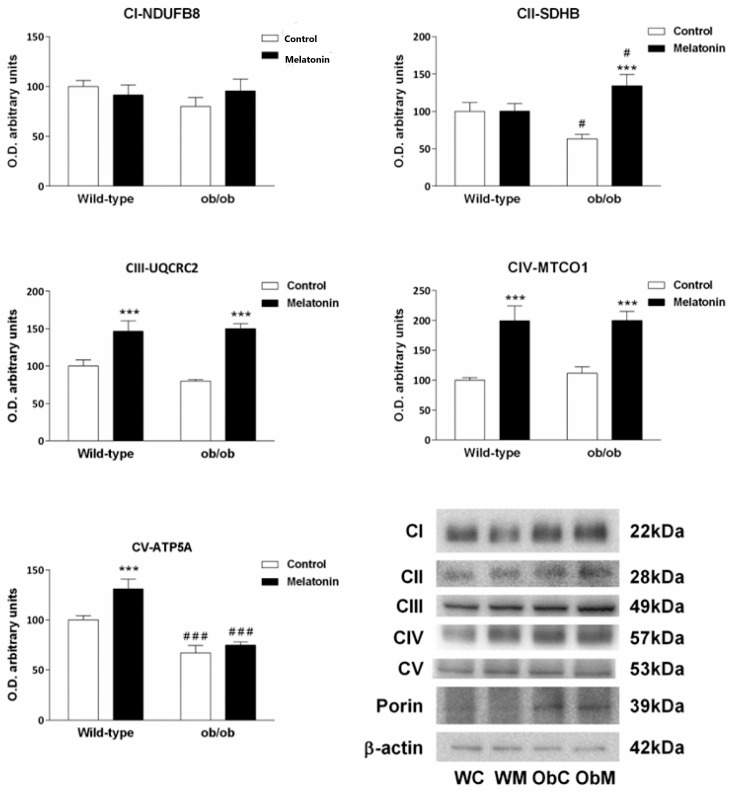
Electron transport chain complexes profile in the livers of wild-type and ob/ob mice. Bar chart showing the semiquantitative optical density (arbitrary units of blot bands) and representative Western blot images of NADH dehydrogenase (ubiquinone) 1 b subcomplex 8 (NDUFB8) from complex I (CI), the iron–sulfur subunit (SDHB) from complex II (CII), the ubiquinol–cytochrome c reductase core protein II (UQCRC2) subunit from complex III (CIII), cytochrome c oxidase subunit I (MTCO1) from complex IV (CIV), and ATP synthase subunit α (ATP5A) from complex V (CV). First, all the immunoblots were normalized to β-actin as a total extract loading control. Then, the complexes were normalized to porin as a mitochondrial mass marker. The data are expressed as the means ± SDs that were calculated from at least three separate measurements. WC—untreated wild-type; WM—wild-type plus melatonin; ObC—untreated ob/ob; ObM—ob/ob plus melatonin. Statistical comparisons: # wild-type vs. ob/ob and * treated with melatonin vs. untreated counterpart. The number of symbols indicates the level of statistical significance: # for *p <* 0.050 and ***/### for *p <* 0.001.

Livers of mice with leptin deficiency presented lower protein expression of the subunits of complexes II and V (SDHB and ATP5A), whereas no effect on the subunits of the other ETC complexes was observed (Figure 4), suggesting that remodeling of the hepatic ETC was associated with leptin deficiency. Melatonin treatment counteracted the effect of leptin deficiency and promoted an increase in the protein content of the subunits of the ETC complexes regardless of the mouse genotype (Figure 4). An exception to the melatonin effect was observed for NDUFB8, the expression of which, in fact, remained constant across all the groups analyzed.

**Figure 5:**
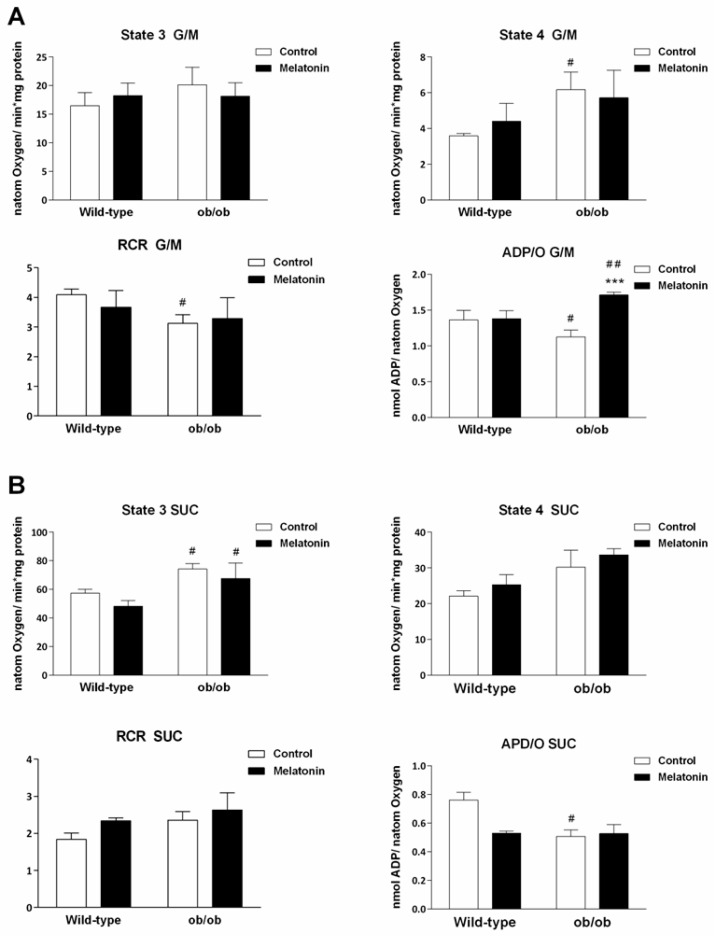
Mitochondrial oxygen consumption was measured with a Clark-type electrode. The energization was obtained with (**A**) glutamate/malate (G/M) and 175 nmol ADP or (**B**) succinate (SUC), rotenone and 125 nmol ADP. State 3 represents the maximum consumption of oxygen in the presence of ADP, and state 4 represents oxygen consumption without ADP consumption (both expressed as nanoatoms of oxygen/min*mg protein). The respiratory control ratio (RCR) determines the coupling between substrate oxidation and oxidative phosphorylation. ADP/O is the efficiency of oxidative phosphorylation and is expressed as nmol ADP/nanoatom of oxygen. The data are expressed as the means ± SDs that were calculated from at least three separate measurements. WC—untreated wild-type; WM—wild-type plus melatonin; ObC—untreated ob/ob; ObM—ob/ob plus melatonin. Statistical comparisons: # wild-type vs. ob/ob and * treated with melatonin vs. untreated counterpart. The number of symbols indicates the level of statistical significance: # for *p <* 0.050, ## for *p <* 0.010, and *** for *p <* 0.001.

To evaluate whether this ETC remodeling implies changes in mitochondrial function, we measured oxygen consumption in mitochondria isolated from the livers of both genotypes. According to the data obtained from state 3 respiration, which is defined as stimulated respiration by exogenously added ADP, no differences in oxygen consumption were noted among the four experimental groups in mitochondria energized with complex I-linked substrates (glutamate/malate, G/M) (Figure 5A). However, when state 4 respiration was analyed, we observed increased respiration rates in the absence of ATP synthesis in ob/ob mice compared with wild-type mice (*p* < 0.050), suggesting greater leak respiration in those animals. Treatment with melatonin did not induce any change in either the state 3 or the state 4 respiration rates, regardless of the genotype (Figure 5A).

As a consequence of increased state 4 respiration, the respiratory control ratio (RCR), calculated as the ratio between states 3 and 4, was significantly lower in ob/ob animals (*p* < 0.050) (Figure 5A), suggesting a decrease in the coupling between substrate oxidation and OXPHOS. Treatment with melatonin did not alter the RCR values. Consistent with increased leak respiration, analysis of the effectiveness of OXPHOS (ADP/O ratio) in the presence of G/M revealed lower values in ob/ob than in wild-type mice (*p* < 0.050). Nonetheless, melatonin treatment increased OXPHOS efficiency in ob/ob animals (*p* < 0.001) (Figure 5A).

With respect to complex II-linked respiration, i.e., succinate (SUC) energization in the presence of rotenone, the state 3 respiration rate was significantly higher in leptin-deficient mice than in wild-type mice (*p* < 0.050). In contrast, no significant differences were detected between strains in terms of the succinate-supported state 4 respiration rate or RCR (Figure 5B). However, a trend toward higher rates of state 4 respiration was detected in ob/ob mice compared with wild-type mice (Figure 5B), explaining the unchanged RCR in this group. The ADP/O ratio in the presence of SUC was significantly lower in ob/ob mice than in wild-type mice (*p* < 0.050). In contrast to complex I-linked respiration, melatonin failed to recover OXPHOS efficiency in the presence of succinate.

### 2.4. Melatonin Restores ATP Production in ob/ob Mice

In view of the results obtained in respiration assays suggesting dysfunction in the ETC and OXPHOS in mitochondria from ob/ob livers and to evaluate whether obesity affects ATP production, we measured 5′ adenosine monophosphate-activated protein kinase (AMPK) activation and basal ATP levels in mitochondrial and cytosolic extracts, as well as the amount of ATP produced after mitochondrial energization with G/M and SUC.

**Figure 6:**
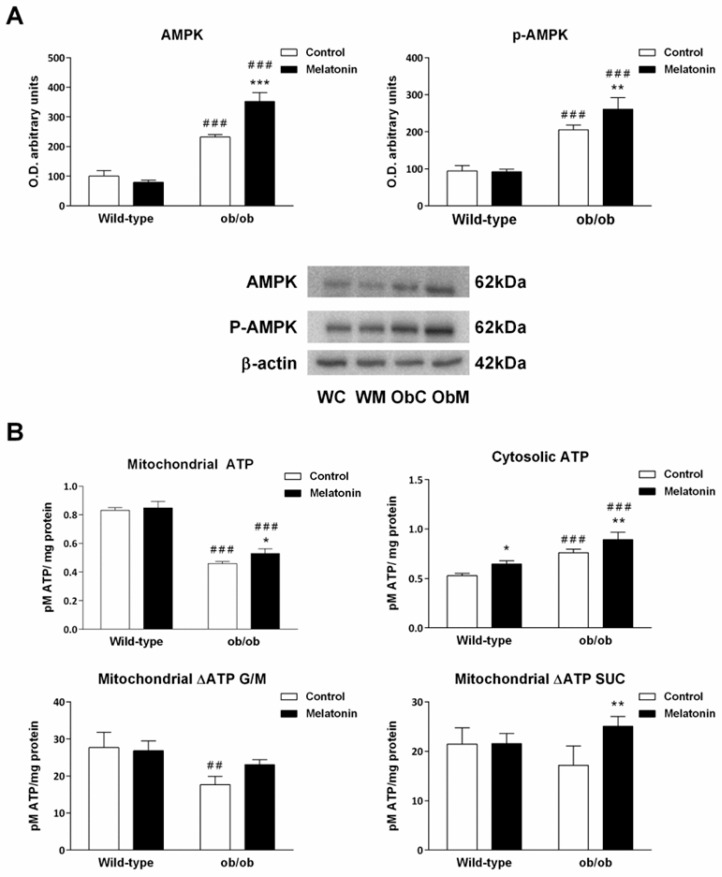
Mitochondrial and cellular energy status. (**A**) Bar chart showing the semiquantitative optical density (arbitrary units of blot bands) and representative Western blot image of adenosine monophosphate-activated protein kinase (AMPK) and its phosphorylated form (p-AMPK). (**B**) Basal mitochondrial and cytosolic ATP content and ATP produced after energization with glutamate/malate (G/M) and succinate (SUC), which was calculated as the difference between the ATP content in mitochondria after respiration and basal mitochondrial ATP. The ATP content is presented as pM ATP/mg protein. The data are expressed as the means ± SDs that were calculated from at least three separate measurements. WC—untreated wild-type; WM—wild-type plus melatonin; ObC—untreated ob/ob; ObM—ob/ob plus melatonin. Statistical comparisons: # wild-type vs. ob/ob and * treated with melatonin vs. untreated counterpart. The number of symbols indicates the level of statistical significance: * for *p <* 0.050, **/## for *p <* 0.010, and ***/### for *p <* 0.001.

AMPK is an important sensor of mitochondrial bioenergetic status [43]. It is activated under conditions in which cellular energy demands are increased and then stimulates ATP production [44]. Both total AMPK and its active phosphorylated form (p-AMPK) were significantly increased in leptin-deficient mice compared with those in wild-type mice (*p <* 0.001) (Figure 6A), suggesting that catabolism in hepatocytes from ob/ob mice is promoted compared with that in hepatocytes from wild-type animals. Supplementation with melatonin had no effect on wild-type mice and further increased AMPK activation in ob/ob mice (*p <* 0.001) (Figure 6A).

Consistent with the results of the respiration experiments, we found that although the mitochondrial ATP levels in the livers of ob/ob animals were lower (*p <* 0.001), the cytosolic ATP content was greater (*p <* 0.001) than that in the livers of wild-type animals (Figure 6B). With respect to melatonin, hormone treatment partially restored ATP levels in the mitochondria of ob/ob mice and increased cytosolic ATP in both ob/ob and wild-type mice (Figure 6B).

The above results suggest a possible effect of leptin on mitochondrial ATP output. Therefore, we next measured ATP synthesis indirectly in mitochondria by calculating the difference between basal mitochondrial ATP and the ATP content in mitochondria at the end of the oxygen consumption experiments. ATP production in mitochondria from ob/ob mice was significantly impaired in the presence of Complex-I-linked substrates but to a lesser degree when Complex-II was used instead (Figure 6B). Overall, melatonin treatment was able to ameliorate ATP synthesis output in both cases but only significantly affected mitochondria energized with SUC.

In summary, melatonin treatment in wild-type mice affected only cytosolic ATP levels. However, in ob/ob mice, melatonin also improved mitochondrial ATP synthesis through an action potentially mediated by AMPK signaling.

### 2.5. Melatonin Reduces the Stimulation of Mitochondrial Fusion Induced by the Absence of Leptin

**Figure 7:**
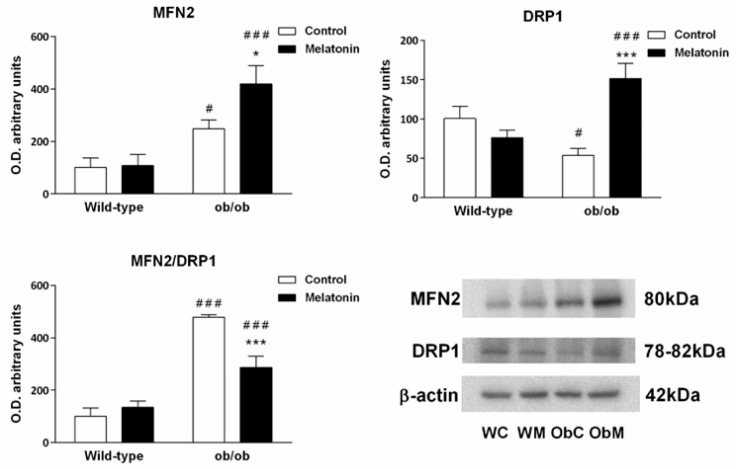
Markers of mitochondrial dynamics in the livers of wild-type and ob/ob mice. Bar charts showing the semiquantitative densitometric analysis (arbitrary units of blot bands) of Western blots for mitofusin 2 (MFN2) and dynamin-related protein 1 (DRP1) normalized to β-actin. Representative immunoblot images and the MFN2/DRP1 ratio are also shown. The data are expressed as the means ± SDs that were calculated from at least three separate measurements. WC—untreated wild-type; WM—wild-type plus melatonin; ObC—untreated ob/ob; ObM—ob/ob plus melatonin. Statistical comparisons: # wild-type vs. ob/ob and * treated with melatonin vs. untreated counterpart. The number of symbols indicates the level of statistical significance: */# for *p <* 0.050, and ***/### for *p <* 0.001.

Mitochondria are very dynamic organelles, and their fusion and fission are fundamental to the regulation of mitochondrial bioenergetics. To evaluate mitochondrial dynamics, we measured the expression of mitofusin 2 (MFN2), a protein involved in mitochondrial fusion, and dynamin-related protein 1 (DRP1), which is involved in mitochondrial fission. The analysis of the data obtained from MFN2 immunoblotting revealed that MFN2 protein expression was significantly increased in the livers of ob/ob mice (*p* < 0.050) (Figure 7). DRP1 expression in the liver followed the opposite pattern and was lower in ob/ob mice than in wild-type animals (*p* < 0.050) (Figure 7). Overall, these findings suggest that mitochondrial fusion processes are activated in leptin-deficient mice.

Melatonin treatment in wild-type animals had no effect on the expression of mitochondrial dynamics-related proteins but upregulated the expression of both proteins in ob/ob mice (*p* < 0.050) (Figure 7). MFN2 and DRP1 are binding partners of each other; therefore, the ratio between them provides useful information about the relative contribution of melatonin to the overall processes of mitochondrial dynamics. Then, we calculated the MFN2/DRP1 ratio and found that melatonin in ob/ob mice potentially prevents the stimulation of mitochondrial fusion observed in obese mice (*p* < 0.001) (Figure 7).

### 2.6. Melatonin Protects against Mitochondrial Outer Membrane Permeabilization in ob/ob Mice

The integrity of the mitochondrial membranes is essential to guarantee correct mitochondrial function. BCL-2 and BAX are proteins that play key roles in mitochondrial outer membrane permeabilization (MOMP), whereas cyclophilin D is the regulatory component of the mitochondrial permeability transition pore (mPTP). Therefore, these three proteins are fundamental for controlling mitochondrial membrane permeability and the subsequent process of mitochondrial apoptosis.

**Figure 8:**
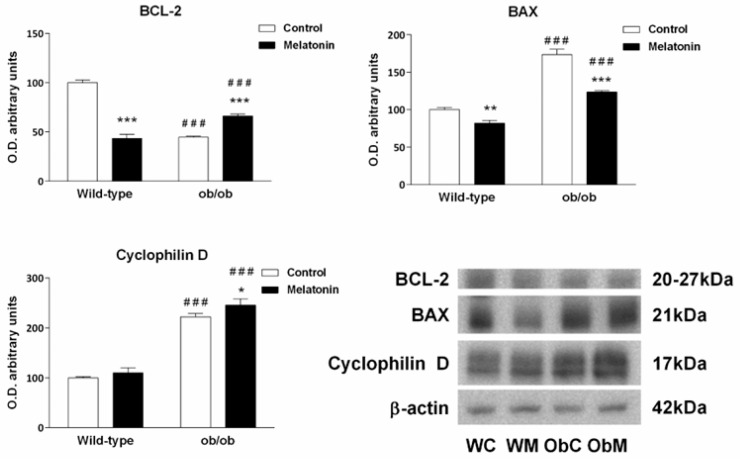
Mitochondrial outer membrane permeabilization markers and cyclophilin D in the livers of wild-type and ob/ob mice. Bar chart showing the semiquantitative optical density (arbitrary units of blot bands) and Western blot images of B-cell lymphoma 2 (BCL2), BCL2-associated X protein (BAX) and cyclophilin D from Western blots normalized to β-actin. The data are expressed as the means ± SDs that were calculated from at least three separate measurements. WC—untreated wild-type; WM—wild-type plus melatonin; ObC—untreated ob/ob; ObM—ob/ob plus melatonin. Statistical comparisons: # wild-type vs. ob/ob and * treated with melatonin vs. untreated counterpart. The number of symbols indicates the level of statistical significance: * for *p <* 0.050, ** for *p <* 0.010, and ***/### for *p <* 0.001.

The evaluation of BCL-2 protein levels revealed that its expression was significantly lower in ob/ob mice than in wild-type mice (*p* < 0.001) (Figure 8). Moreover, the expression of the proapoptotic protein BAX was greater in ob/ob mice (*p* < 0.001) (Figure 8), suggesting that leptin-deficient mice presented a greater MOMP than their wild-type counterparts did. Although melatonin treatment reduced BAX levels in both types of mice (*p* < 0.010), it had opposite effects on the BCL-2 content in ob/ob mice and wild-type mice. Nevertheless, the increased BCL-2 levels found in ob/ob mice after melatonin treatment suggest a protective effect of the hormone in these animals (*p* < 0.001).

Although the identity of the mPTP is debated, cyclophilin D has been accepted as an essential modulator of mPTP under basal and pathological conditions, increasing the probability of the mPTP opening under stress events [45,46]. Our results revealed higher levels of cyclophilin D in ob/ob mice (*p* < 0.001) than in the wild-type mice, suggesting increased sensitivity to the mPTP opening. Surprisingly, melatonin treatment further increased cyclophilin-D expression exclusively in obese mice (Figure 8).

## 3. Discussion

In this study, we explored how the livers of leptin-deficient (ob/ob) mice respond to excessive food intake by initiating harmful cellular processes such as adipogenic steatosis and mitochondrial dysfunction and evaluated the therapeutic effects of melatonin. We previously showed that food intake, body weight at baseline, body weight at sacrifice and body weight changes during testing were significantly greater in ob/ob mice than in wild-type mice. However, melatonin treatment did not affect these weight-related parameters, as no significant differences were observed before and after melatonin administration in mice of the same genotype [47]. Despite these findings, we demonstrated that melatonin, even at low concentrations, can counteract obesity-induced alterations in lipid metabolism and mitochondrial function.

To assess mitochondrial function in the livers of leptin-deficient mice, we measured the protein expression levels of specific subunits of the electron transport chain (ETC) complexes. We observed significantly lower levels of complex II and ATP synthase subunits in ob/ob hepatocytes than in control hepatocytes, indicating structural abnormalities in these components that likely compromise their function and efficiency. Oxygen consumption assays revealed that, compared with those from wild-type mice, the mitochondria from obese mice were uncoupled. This mitochondrial uncoupling negatively impacts the oxidative phosphorylation (OXPHOS) capacity in leptin-deficient mice, as evidenced by decreased ADP/O ratios when both G/M and SUC are used as respiration substrates. Given these indications of mitochondrial dysfunction, we measured the mitochondrial ATP content and found that ob/ob mice produced less ATP than wild-type mice, as reflected by lower basal ATP concentrations and reduced ATP production after G/M and SUC energization. Similar findings were reported by Wang and colleagues [48], who noted that a decrease in ATP production was linked to reduced ATP synthase expression in the livers of diabetic mice. Thus, the diminished ATP synthase expression in leptin-deficient mice is likely a critical factor contributing to their impaired ATP production capacity.

Impaired mitochondrial function is closely associated with excessive reactive oxygen species (ROS) production, potentially exacerbating oxidative stress, as previously described in the livers of leptin-deficient mice [49]. Under physiological conditions, the mitochondrial inner membrane (MIM) is impermeable to most ions and metabolites, maintaining the proton-motive force necessary for ATP production. However, stress conditions, especially those involving increased ROS production and low adenine nucleotide concentrations, can disrupt this permeability barrier [29]. The high of cyclophilin-D expression observed in ob/ob mouse livers suggests that the uncoupling of the ETC and OXPHOS facilitates the opening of the mitochondrial permeability transition pore (mPTP) through cyclophilin-D binding to the MIM. Additionally, the altered expression of BCL-2 family proteins indicates increased mitochondrial outer membrane permeabilization, suggesting a predisposition towards mitochondrial pathways of cell death in hepatocytes from leptin-deficient animals.

AMPK, an energy sensor activated under energy demands to stimulate ATP production, was significantly increased in leptin-deficient mice, confirming that altered energy metabolism demands increased ATP synthesis. Given the increased activation of AMPK in ob/ob mice, the differences in the cytosolic ATP content between leptin-deficient and wild-type mice are notable. The higher cytosolic ATP levels, along with impaired mitochondrial function, suggest an increased reliance on anaerobic glucose metabolism to meet energy demands. The role of leptin in shifting ATP production from glycolytic metabolism to OXPHOS has been documented in MCF-7 cells [50], and the failure of this metabolic switch could explain the elevated cytosolic ATP in ob/ob mice. Moreover, leptin-deficient mice presented increased AdipoR1 and AdipoR2 mRNA expression, indicating low circulating adiponectin levels and defective adiponectin signaling, which have been linked to glucose metabolism deregulation, insulin resistance and diabetes [51,52,53].

Lipid metabolism in leptin-deficient mice was also disrupted, as indicated by increased levels of perilipin, which stabilizes lipid droplets by preventing lipase action. The elevated perilipin content, coupled with reduced lysosomal acid lipase (LAL) expression, suggests decreased lipid turnover and lipolysis, contributing to the accumulation of lipid droplets observed in various obesity models [31,54,55]. LAL deficiency is a recognized cause of dyslipidemia and fatty liver and has been detected in non-alcoholic fatty liver disease patients [56]. Furthermore, increased PPARγ mRNA expression in leptin-deficient mice indicates upregulated adipogenic pathways and adipogenic transformation of hepatocytes. The role of leptin in stimulating lipid oxidation and preventing the progression of steatosis is well-known [57], and the adipogenic steatosis observed in leptin-deficient mice could be attributed to de novo lipogenesis via high glucose/insulin or increased systemic fat uptake, combined with low lipid turnover due to the absence of leptin.

The mitochondrion is crucial for lipid-catabolic and anabolic processes, such as fatty acid β-oxidation and lipogenesis [58], and plays a significant role in the adipogenic process [4]. To understand the bioenergetic alterations in ob/ob mice, we examined mitochondrial biogenesis and dynamics. Increased levels of porin and TFAM, indicators of mitochondrial mass and biogenesis, respectively, were observed in leptin-deficient mice, suggesting that bioenergetic alterations were not due to a lower mitochondrial content. Higher mitochondrial numbers have been previously reported in high-fat diet-fed mice with increased mitochondrial biogenesis but impaired mitochondrial respiration [59]. Accordingly, it has been suggested that the induction of mitochondrial biogenesis by high-fat feeding is beneficial because it increases mitochondrial mass and OXPHOS activity [60]. This action seems to be mediated by AMPK activation, which was also observed in leptin-deficient mice and leads to an increase in NAD+, triggering Sirt1 activation and subsequent PGC-1α induction of mitochondrial biogenesis [44,61,62,63]. These findings suggest that increased mitochondrial biogenesis may be a compensatory response to obesity-related mitochondrial dysfunction.

Mitochondrial fusion and fission are vital for maintaining a healthy mitochondrial network. Fusion allows for the mixing of healthy and damaged mitochondrial proteins, the optimization of resources and the maximization of OXPHOS fidelity [64]. Leptin-deficient mice presented increased levels of the fusion marker MFN2 and decreased levels of the fission-related protein DRP1, indicating a preference for mitochondrial fusion. This adaptive mechanism likely compensates for impaired mitochondrial function. Given the previously reported autophagy blockade in these mice [49], defective organelles might fuse with healthy mitochondria to optimize resources, dilute damage, restore function and prevent intrinsic cell death pathways. Both mitochondrial biogenesis and elongation are crucial during adipocyte differentiation [5], suggesting that increased mitochondrial biogenesis, mass, and fusion in leptin-deficient mice may be related to the adipogenic transformation of hepatocytes.

Despite the absence of significant changes in mitochondrial respiratory parameters, melatonin treatment enhanced OXPHOS efficiency and increased mitochondrial ATP production in the livers of ob/ob mice. Melatonin treatment also remodeled ETC components, including increased levels of the CII-SDHB, CIII-UQCRC2 and CIV-MTCO1 subunits, which do not necessarily increase their activity. Previous studies have shown that higher concentrations of melatonin improve ETC complex assembly and activity in ob/ob mice [11]. This improvement in mitochondrial function likely reduces ROS production, which is consistent with our findings of decreased oxidative stress after melatonin treatment [49,65]. These findings indicate that the effects of melatonin in ob/ob mice are due mainly to its antioxidant properties, which improve mitochondrial function and scavenge free radicals. Additionally, the antiapoptotic effects of melatonin [66,67,68] align with the decreased BAX expression observed after treatment. However, in ob/ob mice, melatonin increases cyclophilin-D levels, which regulate the mPTP [69] and play a role in cell death pathways distinct from those involved in BAX-mediated apoptosis. Cyclophilin-D overexpression is linked to cell death suppression in cancer cells [70] and noncell death-related functions such as mitochondrial calcium efflux during stress [71], increased complex III activity, and supercomplex formation acceleration [72]. Thus, increased cyclophilin-D levels after melatonin treatment might be related to these functions.

Melatonin treatment also elevated cytosolic ATP levels in both strains, possibly due to enhanced mitochondrial function and efficiency, leading to greater ATP export to the cytoplasm or increased glycolytic metabolism.

The treatment of both strains with melatonin reduced AdipoR1 and AdipoR2 mRNA expression, suggesting improved adiponectin signaling and supporting its role in enhancing insulin signaling [73,74,75,76]. Adiponectin/AMPK axis activation has been linked to the promotion of autophagy, alleviation of ER stress and clearance of damaged mitochondria, protecting against steatosis and insulin resistance [77]. We previously reported that melatonin is able to reduce ER stress in the livers of ob/ob mice [49]. Given that melatonin-treated mice presented increased AMPK activation and lower adiponectin receptor levels, melatonin may alleviate the ER stress associated with leptin deficiency through upstream mechanisms.

## 4. Materials and Methods

### 4.1. Animal Model and Treatments

Sixteen 6-week-old male leptin-deficient obese B6.V-Lepob/J (ob/ob) mice and sixteen 6-week-old male wild-type C57BL/6J mice were purchased from Charles River Laboratory (Charles River Laboratories Spain, SA, Barcelona, Spain). The mice were housed two per cage under a 12:12 h dark–light cycle at 22 ± 2 °C and received tap water and a standard chow diet ad libitum.

Unless otherwise stated, all the reagents were purchased from Sigma-Aldrich (Sigma-Aldrich, St. Louis, MO, USA).

After a 2-week acclimatization period, the animals were randomly divided into four groups with eight mice per group: the untreated control groups for wild-type (WC) and ob/ob mice (ObC) and the melatonin-treated groups for wild-type (WM) and ob/ob (ObM) mice. At 2 h after lights off, intraperitoneal injections of melatonin were administered daily at a dose of 500 μg/kg body weight for 4 weeks based on previous studies. Melatonin was dissolved in 0.5% absolute ethanol in saline solution, and the animals in the control groups received vehicle at a comparable dosage, route, and treatment duration.

The animals were sacrificed by decapitation, and the liver of each mouse was removed and either frozen at −80 °C until further use (protein and mRNA analysis) or immediately used for mitochondrial isolation.

The Oviedo University Animal Care and Use Committee approved the experimental protocol. All experiments were performed according to the Spanish Government Guide and the European Community Guide for Animal Care (Council Directive 86/609/EEC).

### 4.2. Western Blot Immunoassay

The frozen livers were homogenized in 1 mL of lysis buffer (50 mM phosphate buffer, pH 7.5; 1 mM NaF; 1 mM Na_3_VO_4_; 1 mM phenylmethylsulfonyl fluoride (PMSF); and 0.1% Triton-X 100) according to methods previously described by our research group [49]. The protein content was determined using the Bradford method [78].

Western blot immunoassays were performed according to previously described protocols [65]. After denaturation at 95 °C for 5 min in Laemmli buffer (161–0737; Bio-Rad, Hercules, CA, USA), equivalent amounts of protein were separated with 12% sodium dodecyl sulfate–polyacrylamide gel electrophoresis (SDS–PAGE) and electrophoretically transferred to a polyvinylidene difluoride (PVDF) membrane. After the membranes were blocked with 5% skim milk in TBS-T (50 mM Tris–HCl, pH 8; 154 mM NaCl and 0.1% Tween-20) for 1 h at room temperature, they were incubated overnight at 4 °C with the following antibodies: AMPK (2793, Cell Signaling, Danvers, MA, USA), p-AMPKα (Thr172) (2535S, Cell Signaling), BAX (2772, Cell Signaling), BCL-2 (sc-7382, Santa Cruz Biotechnology, Dallas, TX, USA), CI-20 (NDUFB8) (ab110242, Abcam, Cambridge, UK), CII-30 (SDHB) (ab14714, Abcam), CIII-Core II (UQCRC2) (ab14745, Abcam), CIV-I (MTCO1) (ab14705, Abcam), CV-a (ATP5A) (ab14748, Abcam), Cyclophilin D (ab110324 (MSA04), Abcam) Drp1 (D6C7) (8570S, Cell Signaling), LAL (ab36597, Abcam), Mfn2 (D2D10) (9482S, Cell Signaling), Perilipin (H-300) (sc-67164, Santa Cruz Biotechnology), Porin (ab14734 (MSA03), Abcam) and β-actin (AC-15, Sigma-Aldrich), each of which were diluted 1:1000 in blocking buffer (1% skim milk in TBS-T). After three cycles of 5 min washes in TBS-T, the membranes were incubated with the corresponding horseradish peroxidase-conjugated secondary antibody (Santa Cruz Biotechnology, Santa Cruz, CA, USA; 1:5000 in blocking buffer) for 2 h at room temperature. After three washes in TBS-T for 5 min each, the membranes were developed with an enhanced chemiluminescence (ECL) detection system (WBKLS0500, Millipore Corporation, Billerica, MA, USA) and imaged with a G:BOX imaging system (Syngene, Cambridge, UK) according to the manufacturers’ protocols.

All the data presented are representative of at least three separate experiments. The density of each band was quantitatively analyzed using Image Studio Lite 3.1 software. The results were normalized to β-actin as a loading control or to Porin as a mitochondrial loading control and are shown in Table S1.

### 4.3. Real-Time RT-PCR

Total RNA was extracted from the livers using the TRI reagent (Sigma Aldrich, St Louis, MO, USA), and cDNA was synthesized with a high-capacity cDNA reverse transcription kit (Invitrogen, Life Technologies, Carlsbad, CA, USA) according to the manufacturers’ protocols. Gene expression was analyzed using real-time PCR with the StepOne Real-Time PCR System (Life Technologies, Carlsbad, CA, USA) following the Applied Biosystems SYBR Green Master Mix protocol (Invitrogen, Life Technologies). The relative messenger RNA (mRNA) expression was calculated using the 2^−ΔΔCT^ method, and the data for each gene in the liver sample were expressed as the fold change normalized to the GAPDH mRNA level and relative to the WC sample. Single PCR products of the correct size were observed in all cases. The primer sequences are shown in Table 1.

### 4.4. Isolation of Mitochondrial and Cytosolic Extracts

The isolation of the mitochondrial and cytosolic extracts was performed following the protocol of Oliveira and Silva [79]. The mouse abdominal cavity was opened, and the liver was removed, placed in liver isolation buffer (LIB: 250 mM sucrose, 10 mM HEPES, 0.5 mM EGTA and 1 mg/mL bovine serum albumin (fatty acid-free), pH 7.4), and cut into small pieces. The suspension was rinsed with LIB to remove all blood and then homogenized with a manual glass Teflon Potter–Elvehjem homogenizer in 7 mL LIB/g liver, with care to maintain a temperature below 4 °C. The homogenate was subsequently centrifuged at 2000× *g* for 10 min at 4 °C, after which the floating fat was carefully aspirated and discarded. The supernatant and the pellet were used to isolate the cytosolic and mitochondrial fractions, respectively.

The supernatant was poured into new refrigerated centrifuge tubes, which were filled with LIB and centrifuged at 12,000× *g* for 10 min at 4 °C. The pellet was discarded, and the supernatant was stored at −80 °C until further use to evaluate the cytosolic fractions.

The pellet from the first centrifugation step was gently resuspended in liver resuspension buffer (LRB) and spun at 12,000× *g* for 10 min at 4 °C. Finally, the pellet representing the mitochondrial fraction was resuspended in 300 μL of LRB. The mitochondria were stored on ice until subsequent experiments or frozen at −80 °C until further use.

### 4.5. Oxygen Consumption

Oxygen consumption assays were performed following the protocol of Oliveira and Silva [79]. Oxygen consumption was measured in a suspension of freshly isolated mitochondria at 37 °C and monitored polarographically with a Clark-type oxygen electrode (Oxygraph, Hansatech Instruments Ltd., Pentney, UK).

The desired volumes (0.5 or 1 mL) of respiration buffer (135 mM sucrose, 65 mM KCl, 5 mM KH_2_PO_4_, 5 mM HEPES, and 2.5 mM MgCl2 (pH 7.4)) and a mitochondrial suspension (1 mg/mL) were introduced into the chamber. Energization was achieved with 10 mM glutamate + 5 mM malate (G/M), which allows entry of electrons into the respiratory chain via Complex I, or with 5 mM succinate (SUC) in the presence of 1 μM rotenone, which feeds electrons to Complex II. In both cases, the phosphorylative system was evaluated by adding 175 or 125 nmol ADP to G/M and SUC, respectively. When all the ADP was consumed, 1 μg of oligomycin was added to determine the degree of oxygen consumption with inhibited ATP synthase.

The oxidation rates were expressed in nanoatoms of oxygen consumed per minute per milligram of protein (nanoatoms oxygen/min×mg protein). The respiratory control ratio (RCR), i.e., the ratio of state 3 to state 4, and the oxidative phosphorylation (OXPHOS) efficiency (ADP/O ratio), i.e., the ratio between the amount of ADP added (in nmol) and the amount of oxygen consumed (in nanoatoms) during state 3, were also calculated.

### 4.6. ATP Levels

The ATP content in the isolated mitochondrial and cytosolic extracts was determined using a commercially available ATP bioluminescent kit (FLAA, Sigma Aldrich). In addition, ATP levels resulting from the phosphorylation of added ADP during oxygen consumption experiments were also evaluated. Measurements were conducted according to the manufacturer’s instructions, with modifications adapted to the conditions of our samples. A standard curve was generated in the range of 74 pM to 600 pM, and a standard or sample previously diluted 1:100 was added to a white 96-well plate (353296, Falcon). Luminescence was measured against the standard curve using a multiplate reader (Synergy HT, BioTek, Winooski, VT, USA). ATP concentrations were expressed as nmol ATP/mg protein.

### 4.7. Statistical Analysis

The statistical software package GraphPad Prism 6 for Windows (GraphPad Software, Inc., La Jolla, CA, USA) was used for all the statistical analyses. The data are presented as the means ± standard deviations (SDs) of the means. The normality of the data was analyzed using the Kolmogorov–Smirnov test. All the data were normally distributed. The effects of melatonin and the genotype were analyzed using a two-way ANOVA, and differences between groups were analyzed using the Bonferroni post-hoc correction. The differences were considered statistically significant when *p* < 0.050.

## 5. Conclusions

**Figure 9:**
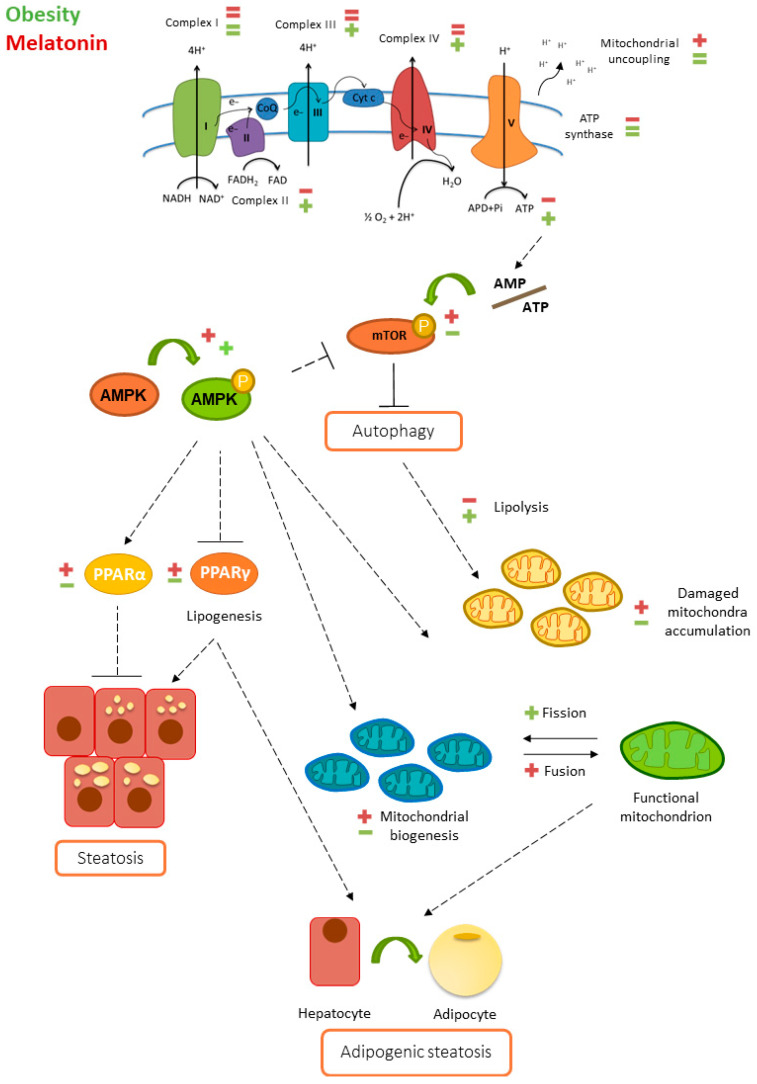
Effect of melatonin on the hepatic mitochondrial adaptations induced by obesity. Obesity (in red) is associated with hepatic mitochondrial dysfunction and lipid accumulation. Thus, obese mice exhibited reduced (minus symbol) levels of some subunits from complex II and complex V, together with decreased ATP production and increased (plus symbol) mitochondrial uncoupling. This mitochondrial energetic status activates the AMPK and adipogenic pathways, contributing to steatosis. Obesity also activates mTOR, suppressing autophagy, which ultimately contributes to the accumulation of damaged mitochondria. The promotion of mitochondrial biogenesis and mitochondrial fusion allows mitochondria to adapt to avoid bioenergetic failure. Melatonin (in green) can partially counteract some of these adaptations by rearranging the electron transport chain machinery, producing relatively high amounts of ATP, inducing mitochondrial turnover mechanisms and attenuating lipogenesis.

This study demonstrated the beneficial effects of short-term melatonin supplementation in countering hepatic mitochondrial alterations associated with leptin deficiency-induced obesity and emphasized the role of this hormone in the reduction of adipogenic pathways in the livers of leptin-deficient mice. Our results indicate that in addition to enhancing lipogenic pathways, leptin deficiency leads to hepatic mitochondrial dysfunction, resulting in reduced mitochondrial ATP production and a metabolic switch to meet energy demands. Increased mitochondrial biogenesis and fusion in ob/ob mice appear to be compensatory mechanisms for dysfunctional mitochondria. Melatonin ameliorates the hepatic mitochondrial adaptations found in ob/ob mice by remodeling the ETC, increasing OXPHOS efficiency and mitigating bioenergetic deficits. In summary, melatonin acts as a mitochondrial protective agent and a direct inhibitor of the adipogenic transformation of hepatocytes, indicating its potential as an agent for interventions in fatty liver disease (Figure 9). In preventive medicine, melatonin could be used to protect mitochondrial function and prevent the progression of hepatic steatosis. In a therapeutic context, melatonin could help reverse mitochondrial dysfunction and improve liver health in patients with fatty liver disease.

## Figures and Tables

**Table 1:** Primer sequences.

	Forward	Reverse
AdipoR1	CCCACCATGCCATGGAGA	GCCATGTAGCAGGTAGTCGTTGT
AdipoR2	CAGGAAGATGAGGGCTTTATGG	GAGGAAGTCATTATCCTTGAGCCA
GAPDH	CAATGACCCCTTCATTGACC	TGGAAGATGGTGATGGGATT
PPARα	TGAAGAACTTCAACATGAACAAG	TTGGCCACCAGCGTCTTC
PPARγ	ACTATGGAGTTCATGCTTGTGAAGGA	TTCAGCTGGTCGATATCACTGGAG
TFAM	ACCTCGTTCAGCATATAACGTTTATGTA	GCTCTTCCCAAGACTTCATTTCAT

## Data Availability

The original contributions presented in the study are included in the article/supplementary material, further inquiries can be directed to the corresponding author/s.

## Supplementary Materials

The following supporting information can be downloaded at: https://www.mdpi.com/article/10.3390/ijms25168677/s1.
